# 419. Changing Epidemiology of Pediatric Invasive Group A *Streptococcus* Infections in the United States, 2004-2022

**DOI:** 10.1093/ofid/ofae631.133

**Published:** 2025-01-29

**Authors:** Veena Ramachandran, Jennifer Okaro, Lesley McGee, Christopher Gregory, Bridget J Anderson, Lee Harrison, Jessica Houston, Ruth Lynfield, Erin Youngkin, Amy Tunali, Ann Thomas, William Schaffner, Susan Petit, Shua Chai

**Affiliations:** Centers for Disease Control and Prevention, Atlanta, GA; Centers for Disease Control and Prevention, Atlanta, GA; Centers for Disease Control and Prevention, Atlanta, GA; Centers for Disease Control and Prevention, Atlanta, GA; New York State Department of Health, Albany, New York; University of Pittsburgh, Pittsburgh, PA; New Mexico Department of Health, Santa Fe, New Mexico; Minnesota Department of Health, St. Paul, MN; Colorado Department of Public Health and Environment, Denver, Colorado; Georgia Emerging Infections Program, Brookhaven, Georgia; Public Health Division, Oregon Health Authority, Portland, Oregon; Vanderbilt University Medical Center, Nashville, Tennessee; Connecticut Department of Public Health, Hartford, Connecticut; California Department of Public Health, Oakland, CA

## Abstract

**Background:**

Recent reports from children’s hospitals of increasing pediatric invasive Group A *Streptococcus* (iGAS) infections triggered national concern. Clindamycin and penicillin dual therapy is often recommended for iGAS, which is life-threatening. We used active, population and laboratory-based surveillance to assess changes in pediatric iGAS disease incidence, clinical outcomes, and bacterial strain characteristics between 2004–2022.

**Methods:**

Using CDC’s Active Bacterial Core surveillance network, we identified pediatric iGAS cases (GAS isolation from a normally sterile site in persons aged ≤17 years) from 2004–2022. CDC *Streptococcus* laboratory characterized available isolates by *emm* typing and antimicrobial susceptibility using conventional and genomic methods. We compared annual incidence during 2004–2019 (pre-COVID-19 pandemic), 2021 (mid COVID-19 pandemic), and 2022 (relaxation of pandemic mitigation measures). Case fatality ratio (CFR), intensive care unit (ICU) admission, and clindamycin non-susceptibility trends (2011-2022) were assessed with the Cochran-Armitage test.

**Results:**

We identified 2,522 pediatric iGAS cases during 2004–2022. Incidence decreased from 1.9 cases per 100,000 persons (2004–2019) to 0.5 (2021) (Incidence Rate Ratio: 0.29 [95% CI: 0.25-0.32]; *P*< .001) but increased in 2022 to 1.5 (2021 vs 2022 IRR: 2.9 [95% CI: 2.6-3.2]; *P*< .001). Thirty-four percent of cases were admitted to the ICU; admission increased from 28.9% (2011) to 36.1% (2022) (*P* = .04). CFR (3.1%) remained stable (P = .69). All isolates were penicillin susceptible; clindamycin non-susceptibility increased from 3.8% (2011) to 28.1% (2021), before declining to 8.4% (2022) (2011 vs 2022 *P* = .01). *emm1*, which accounted for 44.8% of ICU admissions (2011-2022) and 38.5% of fatal cases, decreased to 3.1% of isolates in 2021.

**Conclusion:**

Pediatric iGAS incidence was stable for 15 years before declining substantially in 2021. Infections due to *emm*1, the most identified *emm* type in children with severe disease, almost disappeared in 2021, potentially due to initiation of COVID-19 mitigation measures; however, clindamycin non-susceptibility continued to increase. Rising iGAS incidence in 2022 necessitates close monitoring for rises in *emm*1 infections and antimicrobial resistance.

**Disclosures:**

**Lee Harrison, MD**, GSK: Advisor/Consultant|Merck: Advisor/Consultant|Pfizer: Advisor/Consultant|Sanofi: Advisor/Consultant

